# Fenofibrate Reduces the Severity of Neuroretinopathy in a Type 2 Model of Diabetes without Inducing Peroxisome Proliferator-Activated Receptor Alpha-Dependent Retinal Gene Expression

**DOI:** 10.3390/jcm10010126

**Published:** 2020-12-31

**Authors:** Jennifer M. Enright, Sheng Zhang, Christina Thebeau, Emily Siebert, Alexander Jin, Veda Gadiraju, Xiaodong Zhang, Shiming Chen, Clay F. Semenkovich, Rithwick Rajagopal

**Affiliations:** 1 John F. Hardesty Department of Ophthalmology, Washington University in St. Louis, St. Louis, MO 63110, USA; enrightj@wustl.edu (J.M.E.); zhangsheng@wustl.edu (S.Z.); oberlin.christina@wustl.edu (C.T.); xzhang26@wustl.edu (X.Z.); chenshiming@wustl.edu (S.C.); 2 Department of Pediatrics, UT Southwestern Medical Center, Dallas, TX 75390, USA; esieber4@slu.edu; 3 St. Louis University School of Medicine, St. Louis, MO 63104, USA; alexander.jin@health.slu.edu; 4 University of Washington Medical School, Seattle, WA 98195, USA; vgadiraju@wustl.edu; 5 Division of Endocrinology, Metabolism and Lipid Research, Washington University in St. Louis, St. Louis, MO 63110, USA; csemenko@wustl.edu

**Keywords:** fenofibrate, diabetic retinopathy, PPAR-alpha, electroretinography, Müller glia

## Abstract

Fenofibrate slows the progression of clinical diabetic retinopathy (DR), but its mechanism of action in the retina remains unclear. Fenofibrate is a known agonist of peroxisome proliferator-activated receptor alpha (PPARα), a transcription factor critical for regulating metabolism, inflammation and oxidative stress. Using a DR mouse model, *db/db*, we tested the hypothesis that fenofibrate slows early DR progression by activating PPARα in the retina. Relative to healthy littermates, six-month-old *db/db* mice exhibited elevated serum triglycerides and cholesterol, retinal gliosis, and electroretinography (ERG) changes including reduced b-wave amplitudes and delayed oscillatory potentials. These pathologic changes in the retina were improved by oral fenofibrate. However, fenofibrate did not induce PPARα target gene expression in whole retina or isolated Müller glia. The capacity of the retina to respond to PPARα was further tested by delivering the PPARα agonist GW590735 to the intraperitoneal or intravitreous space in mice carrying the peroxisome proliferator response element (PPRE)-luciferase reporter. We observed strong induction of the reporter in the liver, but no induction in the retina. In summary, fenofibrate treatment of *db*/*db* mice prevents the development of early DR but is not associated with induction of PPARα in the retina.

## 1. Introduction

Diabetes is associated with a high burden of retinal disease, with diabetic retinopathy (DR) affecting over 4 million patients and diabetic macular edema over 1 million patients in the United States alone [1]. Patients with vision-threatening disease may benefit from available treatment options, including vascular endothelial growth factor (VEGF) inhibitors, steroids, pan-retinal photocoagulation, and vitrectomy, but not all patients respond to these therapies [2,3,4]. Furthermore, there is a need for treatments that can slow progression of disease before it becomes vision threatening. Fenofibrate was shown in the Fenofibrate Intervention and Event Lowering in Diabetes (FIELD) and Action to Control Cardiovascular Risk in Diabetes (ACCORD) studies to slow the progression of mild to moderate non-proliferative DR and reduce the need for treatment of macular edema [5,6]. In patients with extant diabetic macular edema, fenofibrate used in conjunction with VEGF inhibitors led to greater improvement than VEGF inhibitors alone [7]. Although fenofibrate shows promise in the treatment of DR, its mechanism of action remains unclear.

Functional data suggest that fenofibrate plays a protective role in mouse and rat models of DR. Electroretinography (ERG) studies of *db/db* mice (a model of type 2 diabetes) at two and five months of age demonstrated reductions in b-wave amplitudes that improved with fenofibrate treatment, and fenofibrate partially rescued a decline in visual function as assessed by optokinetic (OKN) drum in rats treated with streptozotocin (STZ, a model of type 1 diabetes) [8,9,10]. These functional data support the use of animal models in studying the molecular pathways underlying the protective effect of fenofibrate in DR.

Peroxisome proliferator-activated receptor alpha (PPARα) is a master regulator of lipid metabolism, inflammation and anti-oxidation and is a known target of fenofibrate. Although PPARα classically acts in the liver to correct dyslipidemia, the therapeutic effect of fenofibrate in humans was unrelated to circulating lipid levels [5,6]. However, there is evidence suggesting PPARα is directly involved in the pathophysiology of DR. PPARα is expressed in Müller glia in humans and rats, with reduced expression in the setting of diabetes [11]. Further, PPARα appears to be essential for normal retinal function, with PPARα knockout (KO) mice showing reduced b-wave amplitudes, capillary dropout and leakage, increased leukocyte adhesion and increased levels of vascular endothelial growth factor (VEGF), tumor necrosis factor (TNF) and intracellular adhesion molecule (ICAM) [11,12]. These features are worsened in the setting of STZ-induced diabetes [8]. Several protective effects of fenofibrate appear to be dependent on PPARα. Fenofibrate reduced pericyte loss, capillary dropout, and the expression of inflammatory mediators VEGF and ICAM in STZ-induced diabetes, and lessened inflammation and apoptosis in oxygen-induced retinopathy (OIR, a mouse model of retinopathy of prematurity) [13,14,15]. These effects were abrogated in whole-body PPARα null mice. Together, these findings suggest that fenofibrate may act as a PPARα agonist in the retina to moderate DR in a lipid-independent fashion.

The predominant cell type impacted by fenofibrate also remains an open question. The retina is a heterogeneous tissue comprised of multiple neuronal, glial and vascular subtypes. Rod photoreceptors constitute 80% of the cell population in the mouse and rat retina, so studies of whole retina such as Western blot and qPCR largely reflect changes in rod photoreceptors. However, other cell types are likely involved in the pathogenesis of DR. In particular, Müller glia play a key role in metabolism, express PPARα at baseline, and undergo gliosis in response to numerous stressors including diabetes [16,17,18,19]. Gliosis can be reversed with fenofibrate, and expression of GLAST (a key glutamate transporter) is rescued by fenofibrate treatment in *db/db* mice [10]. The Müller glia in mice with STZ-induced diabetes demonstrated an upregulation of oxidative stress response genes (*Nrf2*, *Nqo-1 Ho-1* and *Sod1*), which was enhanced by fenofibrate. Fenofibrate also inhibited the upregulation of the nlrp3 inflammasome, IL-1β and caspase-1 in the Müller glia of STZ-treated mice. Therefore, Müller glia are potential direct targets of fenofibrate in the retina.

Here, we further characterized the protective effect of fenofibrate in the *db/db* model of type 2 diabetes, with a focus on metabolism, gliosis and ERG changes. In addition, we tested our hypothesis that fenofibrate acts as a PPARα agonist, directly targeting the retina and Müller glia. We profiled global gene expression changes in non-diabetic, whole mouse retinas treated with oral or intravitreous fenofibrate, assessed the expression of PPARα target genes in Müller glia, and tested the capacity of the retina to respond to PPARα activation using a luciferase reporter.

## 2. Materials and Methods

### 2.1. Mice

Mouse husbandry and procedures, including euthanasia, complied with the Guide for the Care and Use of Laboratory Animals of the National Institutes of Health and were approved by the Washington University in St. Louis Institutional Animal Care and Use Committee (Protocol # 19-0950). Mouse strains included C57BL/6J (The Jackson Laboratory, Stock #00664), BKS.Cg-*Dock7^m^* +/+ *Lepr^db^*/J (“*db/db*”, The Jackson Laboratory, Stock #00642)*,* Tg(Slc1a3-cre/ERT)1Nat/J (“GLAST-CreER”, The Jackson Laboratory, Stock #012586), and B6.Cg-*Gt(ROSA)26Sor^tm14(CAG-tdTomato)Hze^*/J (“tdTomato” The Jackson Laboratory, Stock #007914) [20,21,22]. For in vivo luciferase assays, we used a peroxisome proliferator response element luciferase transgenic mouse on the C57/BL/6J background (repTOP PPRE-Luc, Charles River), as previously described [23,24]. After weaning, all mice were maintained on a standard chow diet until 3 months of age. At 3 months, mice were maintained on standard chow or transitioned to a custom-milled standard chow diet supplemented with 0.2% *w/w* fenofibrate (Envigo Teklad rodent diet) as previously described [25].

### 2.2. Metabolic Analyses

Mice underwent a six-hour fast on hardwood bedding prior to performing serum analyses. Samples from the tail vein were tested for serum glucose using reagents from Sigma Pharmaceuticals (North Liberty, IA, USA). Serum triglyceride and cholesterol measurements were made using reagents from Thermo Fisher Scientific (Waltham, MA, USA). Free fatty acids (FFAs) were assayed using reagents from Wako (FUJIFILM Wako Chemicals U.S.A., Richmond, VA, USA). For total cholesterol measurements from liver and retina, freshly isolated tissue was weighed and homogenized in a 1.5 mL mixture of chloroform and methanol (2:1 *v/v*). The homogenate was centrifuged at 13,400× *g* for 10 min at 4 °C. One hundred μL of the upper organic phase was evaporated in a 1.5 mL tube at room temperature for 30 min. Once dried, 100 μL of the cholesterol detection reagent (Infinity Total Cholesterol reagent, cat# TR13421, Thermo Fisher Scientific (Waltham, MA, USA)) was added to the tube and allowed to incubate at room temperature for 30 min. The mixture was transferred to a 96-well plate, and absorbance at 490 nm was determined. Absolute quantification was achieved by comparing results to a standard curve, and all measurements were normalized to input retinal mass.

### 2.3. Electroretinography

At 6 months of age, mice treated with fenofibrate-supplemented chow and age-matched mice maintained on standard chow underwent electroretinography using the UTAS BigShot System (LKC Technologies, Inc., Gaithersburg, MD, USA) as previously described [26]. Briefly, mice were dark adapted overnight, then anesthetized with ketamine (80 mg/kg total body mass) and xylazine (15 mg/kg lean body mass). Pupils were dilated with 1% atropine sulfate. Full-field white light flashes (10 μs) in darkness or in dim background illumination (30.0 candela/m^2^) were used as the stimulus, with 10 repeats used for the dimmest flashes, and 5 for the brightest. Repeated trials were averaged for each flash luminance. Using MATLAB software (MathWorks, Natick, MA, USA), the amplitude of the a-wave was measured from the average pretrial baseline to the most negative point of the average trace, and the b-wave amplitude was measured from that point to the highest positive point, after subtracting oscillatory potentials (OPs). The OPs were isolated using a digital Butterworth 25 Hz high-pass filter. The eye with the larger b-wave wave amplitude was used for each mouse. The log luminance of the stimulus was calculated based on the manufacturer’s calibrations.

### 2.4. Immunohistochemistry

Whole eyes were isolated from euthanized mice and fixed overnight in 4% paraformaldehyde. The tissue was dehydrated, cryopreserved in sucrose and embedded in optimal cutting temperature (OCT) compound. A Shandon Cryotome E cryostat was used to prepare 15-micron sections through the optic nerve head. Sections were permeabilized with 0.5% Triton X-100/PBS and then incubated overnight at 4 °C with primary rabbit antibodies against glial fibrillary acidic protein (GFAP; Agilent Technologies (Santa Clara, CA, USA), catalog #Z033429-2) at 1:500 dilution in 10% donkey serum/0.5% Triton X-100/PBS. Sections were then rinsed in PBS and incubated with secondary antibodies (conjugated to Alexa Fluor 594; Thermo Fisher Scientific (Waltham, MA, USA)) under the same conditions. Sections were then mounted in a medium containing DAPI (Dako Fluorescence Mounting Medium, Agilent Technologies (Santa Clara, CA, USA)). Single field light microscopy images were acquired with a Nikon Eclipse Ti inverted microscope (Nixon, Minato City, Tokyo, Japan) coupled to an LED light source (Lumencor, Beaverton, OR, USA) using a 40× (N.A. 1.4) objective and processed with the NIS element software package. Relative fluorescence from acquired images was measured using ImageJ software (National Institutes of Health, Bethesda, MD, USA) and were processed using Adobe Illustrator (Adobe Inc., San Jose, CA, USA).

### 2.5. Gene Expression Analysis of Mice Treated with Fenofibrate-Supplemented Diet

Whole retina was isolated from eight 6-month-old C57BL/6J mice receiving a fenofibrate-supplemented diet, and eight sibling controls (with four male and four females per group). RNA was isolated using Trizol, RNA concentration was determined by Qubit, and RNA quality was assessed with an Agilent 2100 Bioanalyzer Total RNA nano chip (Agilent Technologies Inc., Santa Clara, CA, USA). Library preparation was performed with 1 ug of total RNA. Ribosomal RNA was removed by a hybridization method using the Ribo-ZERO kit (Illumina, San Diego, CA, USA). Depletion and mRNA yield were confirmed with an Agilent 2100 Bioanalyzer as above. Messenger RNA was then fragmented in buffer containing 40 mM Tris Acetate pH 8.2, 100 mM Potassium Acetate and 30 mM Magnesium Acetate and heating to 94 °C for 150 s. Messenger RNA was reverse transcribed to yield cDNA using SuperScript III RT enzyme (per manufacturer’s instructions, Life Technologies, Carlsbad, CA, USA) and random hexamers. A second strand reaction was performed to yield ds-cDNA. Complimentary DNA was blunt ended, had an A base added to the 3′ ends, and then had Illumina sequencing adapters ligated to the ends. Ligated fragments were then amplified for 12–15 cycles using primers incorporating unique index tags. Libraries were quantified using Qubit assays and quality assessed on an Agilent TapeStation system (Agilent Technologies, Santa Clara, CA, USA). An equimolar pool was made of all libraries with unique indices. Fragments were sequenced on an Illumina HiSeq-3000 using single reads extending 50 bases (Illumina, San Diego, CA, USA).

Basecalls and demultiplexing were performed with Illumina’s bcl2fastq software and a custom python demultiplexing program with a maximum of one mismatch in the indexing read (Illumina, San Diego, CA, USA). RNA-seq reads were then aligned to the Ensembl release 76 top-level assembly with STAR version 2.0.4b [27]. Gene counts were derived from the number of uniquely aligned unambiguous reads by Subread:featureCount version 1.4.5 [28]. Sequencing performance was assessed for the total number of aligned reads, total number of uniquely aligned reads, and features detected. The ribosomal fraction, known junction saturation, and read distribution over known gene models were quantified with RSeQC version 2.3 [29].

All gene counts were then imported into the R/Bioconductor package DESeq [30] and trimmed mean of m-values (TMM) normalization size factors were calculated to adjust for differences in library size. Genes or transcripts not expressed in any sample were excluded from further analysis. Differential expression analysis was then performed to analyze for differences between conditions and the results were filtered for only those genes with Benjamini–Hochberg false-discovery rate adjusted *p*-values less than or equal to 0.05.

### 2.6. Intravitreous Injection and Microarray Analysis

Twelve male, 3-month-old C57BL/6J mice were anesthetized with ketamine (80 mg/kg total body mass) and xylazine (15 mg/kg lean body mass). Three mice each received bilateral intravitreous injection of 1 μL fenofibrate 250 μM in 50% DMSO in normal saline (NS), 1 μL GW590735 500 nM in 50% DMSO/NS, 1 μL WY14643 60 μM in 50% DMSO/NS, or 1 μL 50% DMSO/NS as a vehicle control [15,31]. Injections were performed using a 32-gauge needle introduced dorsally into the vitreous cavity just posterior to the limbus and directed away from the crystalline lens. Topical antibiotic ointment was applied to each eye and all mice were allowed to recover on a warming pad.

After 24 h, whole retina was dissected from euthanized mice. The two retinas from each animal were pooled, and RNA was isolated using Trizol. RNA quality was assessed using an Agilent 2100 Bioanalyzer Total RNA nano chip (Agilent Technologies Inc., Santa Clara, CA, USA). All samples had a RNA integrity number (RIN) score > 8.0. Amplified cDNA libraries were generated using the NuGEN Ovation PicoSL kit (NuGEN Technologies Inc., Redwood City, CA, USA), and hybridized to a GeneChip Mouse Gene 2.0 ST Array (Affymetrix Inc., Santa Clara, CA, USA). Data were analyzed using the Affymetrix Expression Console software.

### 2.7. Isolation and Gene Expression Analysis of Müller Glia

Tamoxifen was used to induce transgene expression in GLAST-*CreER+*; R26CL*:tdTomato fl/fl* mice and GLAST-*CreER(-/-)*; R26CL*:tdTomato fl/fl* siblings at age 1–2 months. Tamoxifen (Sigma-Aldrich Inc., St. Louis, MO, USA) was reconstituted in ethanol, and diluted in sunflower oil at a concentration of 20 mg/mL. Intraperitoneal injections of tamoxifen 1–2 mg were then carried out for 5 consecutive days.

At six months of age, retinas were harvested and Müller glia isolated using fluorescence-activated cell sorting (FACS), as previously described [32]. Briefly, retinas were dissected into calcium- and magnesium-free PBS and dissociated with 1 mg/mL trypsin (Sigma-Aldrich Inc., St. Louis, MO, USA) and mechanical trituration. The reaction was then inhibited with 1 mg/mL trypsin inhibitor and suspended cells were treated with DNase I (Sigma-Aldrich Inc., St. Louis, MO, USA). Cells were sorted based on fluorescence in the PE-A channel on a FACS Aria II (BD Biosciences, San Jose, CA, USA).

RNA was isolated using the RNeasy micro kit (QIAGEN Inc., Venlo, The Netherlands) and samples were stored at −80 °C. Reverse transcription with Superscript II (Invitrogen Inc., Carlsbad, CA, USA) was carried out using the standard manufacturer protocol on a Bio-Rad thermocycler. Quantitative PCR (qPCR) was performed using a StepOnePlus Real-Time PCR System (Thermo Fisher Scientific Inc., Waltham, MA, USA) with SYBR green reagents and the qPCR primers listed in Table 1, with two technical replicates per sample. Statistical analysis was conducted in Microsoft Excel (Microsoft Inc., Redmond, CA, USA), using an unpaired Student’s *t*-test.

### 2.8. Luciferase Assay

Transgenic mice carrying the PPRE-luciferase reporter [23,24] underwent intraperitoneal (IP) delivery of 10 mg/kg GW590735 or intraocular delivery of 2 μL of 500 nM GW590735 at 3 months of age. Retina and liver tissue were then harvested 24 h later. A Promega Dual-Luciferase Reporter system (Promega, Madison, WI, USA, catalog #E1910) was used to perform luciferase assays. Briefly, harvested tissues were weighed and washed in PBS. For both retina and liver, a total of 10 mg tissue (wet weight) was homogenized with a tissue grinder and incubated with 0.5 mL passive lysis buffer (PLB, Promega, Madison, WI, USA, catalog #194A1) for 15 min. After a brief low speed centrifugation to pellet the cellular debris, 10 μL of the supernatant was mixed with 50 μL of assay buffer containing the D-luciferin reaction substrate. Light emission from the mixture was then analyzed using a Turner TD-20/20 luminometer and emission spectra corresponding to firefly and *Renilla* luciferase were recorded. Readings from the *Renilla* channel were subtracted from the firefly channel to remove background noise. Luminance readings were analyzed relative to the vehicle control.

### 2.9. Statistical Analyses

Distributions of electroretinography responses across groups at different light intensities are described by means ± SEM. Sample size was at least *n* = 3 per experimental group and was often larger, as indicated in the legends of specific results figures. Statistical analyses were performed using GraphPad 6 Prism software. As indicated in specific figure legends, ordinary two-way ANOVA, ordinary 1-way ANOVA, or Student’s *t*-test was used to compare groups within experiments. Post-hoc multiple comparisons tests were performed using Bonferroni’s correction. All analyses were performed with significance at *p* < 0.05.

## 3. Results

### 3.1. Oral Fenofibrate Alters Lipid Metabolism in a Mouse Model of Type 2 Diabetes

We assessed the effect of fenofibrate on metabolism in the *db/db* mouse model of type 2 diabetes. The leptin-receptor mutant *db/db* mouse develops a number of metabolic abnormalities, including obesity, altered lipid profile, and diabetes, all of which are exacerbated by a high-fat diet [22,33]. These global metabolic changes are comparable to those seen in human patients with type 2 diabetes. At three months of age, *db/db* mice and their heterozygous *db/+* littermates were randomized to receive either chow supplemented with 0.2% *w/w* fenofibrate, or standard chow for the subsequent three months. At baseline, *db/db* mice were heavier and had elevated serum glucose relative to their littermates (Table 2). There was no difference in these parameters between mice randomized to receive fenofibrate or standard chow.

At six months of age, after three months of treatment, further metabolic studies were conducted. At this timepoint, *db*/*db* mice remained heavier and had higher serum glucose than their littermates, regardless of fenofibrate supplementation. Plasma triglycerides were markedly elevated in *db/db* mice that remained on regular chow. However, this elevation was abrogated in *db/db* mice with fenofibrate supplementation. Plasma free fatty acids were elevated in *db/db* mice. There was no significant difference in free fatty acids between *db/db* mice on fenofibrate-supplemented and standard chow, although there was a trend for lower levels when fenofibrate was given. Plasma cholesterol was elevated in *db/db* mice relative to their littermates. Fenofibrate supplementation led to further increase in plasma cholesterol in *db/db* and *db/+* animals.

To further explore the elevated serum cholesterol levels observed with fenofibrate supplementation, we measured cholesterol levels in retina and liver (Table 3). Cholesterol levels in the liver were elevated in *db/db* mice relative to littermate controls. However, liver cholesterol was not altered by fenofibrate treatment in either *db/db* or *db/+* animals. Cholesterol levels in the retina were unaffected by genotype or fenofibrate supplementation. Therefore, while serum cholesterol is elevated by fenofibrate supplementation, fenofibrate does not alter cholesterol content of the liver or retina in wild type or *db/db* mice.

### 3.2. Diabetes-Associated Reactive Retinal Gliosis Is Reduced by Oral Fenofibrate

**Figure 1:**
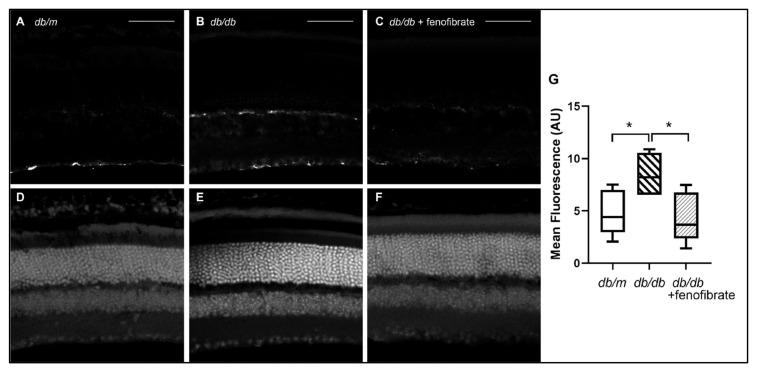
**Fenofibrate reduces diabetes-associated reactive retinal gliosis.** Retinas were harvested from *db/db* mice and their heterozygous siblings at six months of age, fixed, sectioned and labeled with glial fibrillary acidic protein (GFAP) or 4′,6-diamidino-2-phenylindole (DAPI). (**A**) Heterozygous *db/+* mice show minimal GFAP staining localized to the internal limiting membrane. (**B**) Diabetic *db/db* mice showed evidence of gliosis, with GFAP expression localized to the inner nuclear layer (INL) as well. (**C**) Oral fenofibrate treatment of *db/db* mice abrogated gliosis, with GFAP staining appearing comparable to that of *db/+* siblings. (**D**–**F**) DAPI was used as a counterstain to demonstrate nuclear morphology. Scale bars represent 50 microns. (**G**) Quantification of relative fluorescence signal between experimental groups (*n* = 6/group). * = *p* < 0.05, one-way ANOVA.

Gliosis is a reactive change in the morphology of Müller glia occurring subsequent to a variety of inflammatory and ischemic insults including DR. It is characterized by an increased elaboration of intracellular processes and increased expression of glial fibrillary acid protein (GFAP). Using immunohistochemistry targeting GFAP, we observed increased gliosis in six-month-old *db/db* mice relative to their *db/+* littermates (Figure 1A,B). However, gliosis was not observed in *db/db* mice maintained on fenofibrate-supplemented diet (Figure 1C). Retinal morphology was grossly intact in all groups based on DAPI nuclear counterstain (Figure 1D–F).

### 3.3. Oral Fenofibrate Attenuates Diabetes-Related Changes in Electroretinography

**Figure 2:**
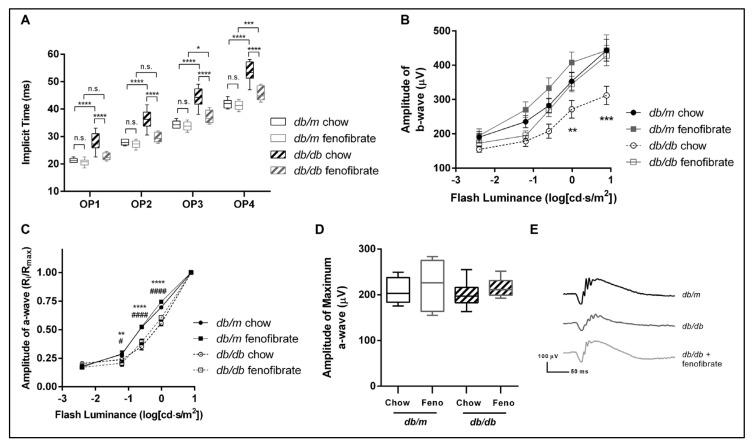
**Oral fenofibrate attenuates diabetes-related changes in electroretinography.** Diabetic *db/db* mice and their heterozygous *db/+* siblings were maintained on a fenofibrate-supplemented diet or standard chow and underwent electroretinography (ERG) at six months of age. (**A**) The implicit time for the first four oscillatory potentials (OP1–OP4) is shown. A statistically significant delay of all four oscillatory potentials occurs in *db/db* mice maintained on regular chow relative to littermate controls. This delay was attenuated in *db/db* mice maintained on fenofibrate-supplemented diets. (**B**) B-wave amplitude was measured at a range of flash luminance. Significantly reduced b-wave amplitudes at higher luminance values were observed in *db/db* mice fed regular chow, but not in *db/db* mice given fenofibrate. (**C**) A-wave amplitudes across a range of flash luminance are shown. A-wave reduction was noted at some levels of luminance for *db/db* mice regardless of diet. (**D**) No differences in the maximum a-wave amplitude were observed between the four groups. *n* = 10 per group. * = *p* < 0.05, ** = *p* < 0.01, *** = *p* < 0.001, **** = *p* < 0.0001, two-way ordinary ANOVA, compared to healthy control within same treatment group. # = *p* < 0.05, #### = *p* < 0.0001, two-way ordinary ANOVA, compared to genotype-matched control in the opposite treatment group. (**E**) Illustrative scotopic electroretinograms from a −0.01 log(cd⋅s/m^2^) flash.

Retinal function in *db/db* and *db/+* mice was assessed at six months of age using ERG to assess oscillatory potential (OP) implicit times, along with a-wave and b-wave amplitudes in dark-adapted animals. The OP implicit time is thought to be indicative of inner retinal function and may be driven by amacrine cells. We found that the OP implicit time was delayed in *db/db* mice relative to their littermate controls (Figure 2A). Further, we found that fenofibrate partially rescued the delay in OP implicit time in *db/db* mice. Similarly, b-wave amplitude reduction was observed in *db/db* mice receiving regular chow, but not in the setting of fenofibrate supplementation (Figure 2B). A-wave amplitudes were reduced at some luminance levels in *db/db* mice regardless of fenofibrate treatment, although maximum a-wave amplitude was similar between all groups (Figure 2C,D). Together, these data suggest that inner retinal dysfunction induced by a diabetic state can be tempered by fenofibrate, and that improvement in amacrine cell function may be a contributing factor. However, outer retinal function does not appear to be affected by fenofibrate at this time point.

### 3.4. Oral Fenofibrate Does Not Alter Expression of PPARα Targets in Whole Retina of Non-Diabetic Mice

**Figure 3:**
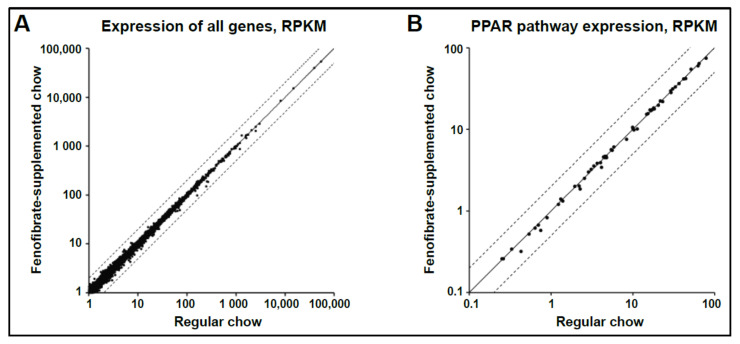
**Gene expression is unchanged in whole retinas isolated from mice treated with a fenofibrate-supplemented diet.** Beginning at one month of age, mice were maintained on a fenofibrate-supplemented or regular chow diet, with four male, and four female mice per group. They were harvested at six months of age. RNA was isolated from whole retinas and gene expression assessed using RNA-seq. Each point represents the normalized expression of a single gene in reads per kilobase per million mapped reads (RPKM). The solid line represents equal expression in the two groups, while the dashed lines represent a 2-fold difference in gene expression. (**A**) Gene expression in all genes. (**B**) Gene expression in the subset of genes belonging to the PPAR pathway (as annotated in KEGG).

Given the changes in metabolism, gliosis and retinal function elicited by fenofibrate treatment of diabetic mice, we hypothesized that oral fenofibrate alters gene expression in the mouse retina. To assess global changes in gene expression, whole retina was isolated from six-month-old, wild-type C57BL/6J mice that had been maintained on a regular or fenofibrate-supplemented diet. RNA was extracted and subjected to high-throughput sequencing, followed by differential gene expression analysis. Gene expression was remarkably similar between the two treatment groups, with no genes showing significant changes after multiple comparisons correction (Figure 3A). As fenofibrate is a known inducer of PPARα, we looked specifically at genes annotated as belonging to the PPAR pathway in the Kyoto Encyclopedia of Genes and Genomes (KEGG) database. Again, there were no significant changes (Figure 3B).

### 3.5. Intravitreous Fenofibrate Does Not Alter Expression of PPARα Targets in Whole Retina of Non-Diabetic Mice

**Figure 4:**
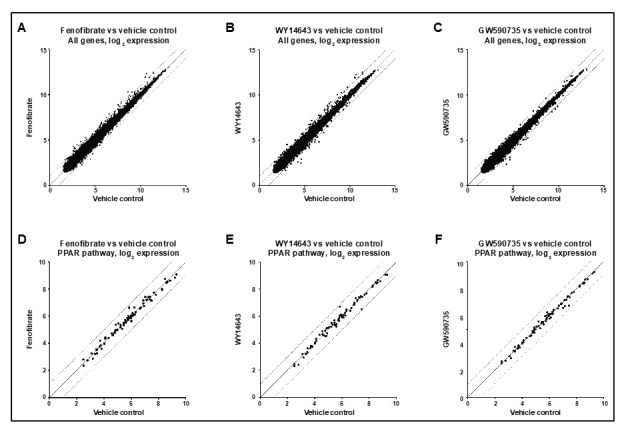
**Gene expression is unchanged in whole retinas isolated from mice treated with intravitreous fenofibrate or known PPARα pathway activators.** The vitreous cavities of both eyes of three-month-old male mice were injected with fenofibrate, GW590735, WY14643 or a vehicle control (*n* = 3). Whole retinas were harvested 24 h later and RNA was isolated, amplified and analyzed by microarray. (**A**–**C**) Log2 expression levels for all probes in the drug-treated vs. control animals are shown for fenofibrate, GW590735, and WY14643. (**D**–**F**) The subset of probes corresponding to genes belonging to the PPAR pathway (as annotated in KEGG) are shown. The solid line represents equal expression in the two groups, while the dashed lines represent a 2-fold difference in gene expression.

We next explored if local delivery of fenofibrate would be more efficacious than oral supplementation in inducing changes in gene expression in the retina. Three-month-old male mice underwent intravitreous injection of fenofibrate (1 μL, 250 μM) or injection with DMSO/normal saline (1 μL, 50:50) as a vehicle control. Whole retina was obtained 24 h later, RNA was isolated and transcript levels globally assessed using a microarray. Similar to oral supplementation, gene expression was remarkably similar between the animals injected with fenofibrate and vehicle control, with no genes showing significant changes after multiple comparisons correction (Figure 4A). In particular, genes involved in the PPAR pathway (per KEGG database) showed stable levels of expression (Figure 4D). Two additional PPARα agonists were also tested as positive controls. GW590735 (1 μL, 500 nM) and WY14643 (1 μL, 60 μM) were similarly injected into the vitreous of three-month-old mice. Microarray analysis revealed relatively stable gene expression globally, and in PPAR pathway genes specifically (Figure 4B,C,E,F).

### 3.6. Expression of PPARα Targets Is Unchanged in Müller Glia Isolated from Mice Treated with Oral Fenofibrate

As whole retina is largely comprised of rod photoreceptors, populations of rarer cell types such as Müller glia must be enriched in order to study their gene expression. To specifically test whether the PPARα pathway is activated by fenofibrate in Müller glia, these cells were isolated using GLAST*-CreER+*; R26CL*:tdTomato fl/fl* mice, in which tdTomato is stably expressed in glial cells following treatment with tamoxifen. Fluorescence-activated flow cytometry (FACS) was then used to generate cell populations enriched for Müller glia based on their red fluorescence.

**Figure 5:**
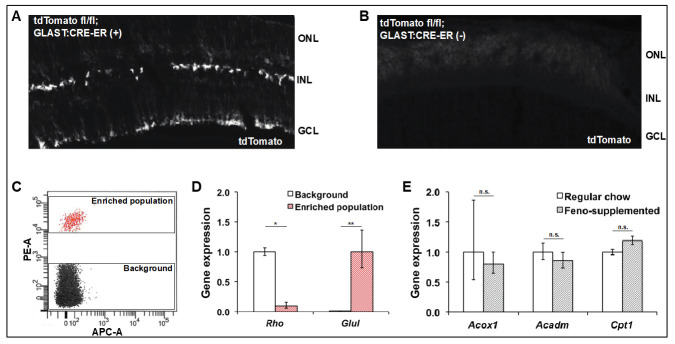
**PPARα target genes are not upregulated in Müller glia isolated from mice treated with a fenofibrate-supplemented diet.** (**A**) When GLAST-CreER; R26CL*-tdTomato* transgenic mice are induced with tamoxifen, robust expression of tdTomato is noted in the Müller glia. (**B**) while no tdTomato expression is seen in the retinas of mice lacking the GLAST-CRE-ER transgene. (**C**) GLAST-CreER and R26CL-*tdTomato fl/fl* transgenic mice were started on a fenofibrate-supplemented diet or kept on regular chow at one month of age, and were then induced with tamoxifen. At six months of age, the retinas were harvested, dissociated, and tdTomato+ cells were isolated using fluorescence activated cell sorting (FACS). (**D**) The rod marker rhodopsin was depleted in the sorted population, while glutamine synthetase, a Müller glia marker, was significantly enriched (*n* = 4, Student’s *t*-test). (**E**) There was no difference in the expression of the PPARα targets *Acox1*, *Acadm*, and *Cpt1a* in enriched Müller glia populations isolated from mice treated with fenofibrate-supplemented diet or regular chow (*n* = 3, Student’s *t*-test). ONL (outer nuclear layer), INL (inner nuclear layer), GCL (ganglion cell layer). * = *p* < 0.05, ** = *p* < 0.01.

Müller glia enrichment was assessed using GLAST*-CreER+*, R26CL*:tdTomato fl/fl* and their GLAST-CreER negative littermates. These mice were induced with tamoxifen at 1–2 months of age, and retinas harvested at six months of age. Frozen sections confirmed induction of tdTomato in a pattern consistent with Müller glia in CRE(+) animals, but not their CRE(−) littermates (Figure 5A,B). FACS of dissociated retina isolated from CRE(+) animals revealed a fluorescent cell population, which was collected along with a non-fluorescent background cell population (Figure 5C). Using qPCR, we found decreased expression of rhodopsin transcript in the tdTomato+ population, suggesting a depletion of rod photoreceptors, and a concomitant increase in the Müller marker glutamine synthetase (Figure 5D).

The effect of oral fenofibrate on gene expression in Müller glia was then determined by maintaining GLAST*-CreER+*; R26CL*:tdTomato fl/fl* mice on either regular chow or a fenofibrate-supplemented diet. These mice were induced with tamoxifen at 1–2 months of age. Retinas were isolated at six months of age and FACS was used to isolate Müller-enriched populations from both groups. Known PPARα-target genes, *Acox1*, *Acadm,* and *Cpt1*, were not induced in mice treated with fenofibrate, as determined using qPCR (Figure 5E). These data suggest that oral delivery of fenofibrate does not activate PPARα in Müller glia in the non-diabetic state.

### 3.7. Systemic or Local Delivery of a PPARα Agonist Activates a PPRE-Luciferase Reporter in Liver but Not Retina

**Figure 6:**
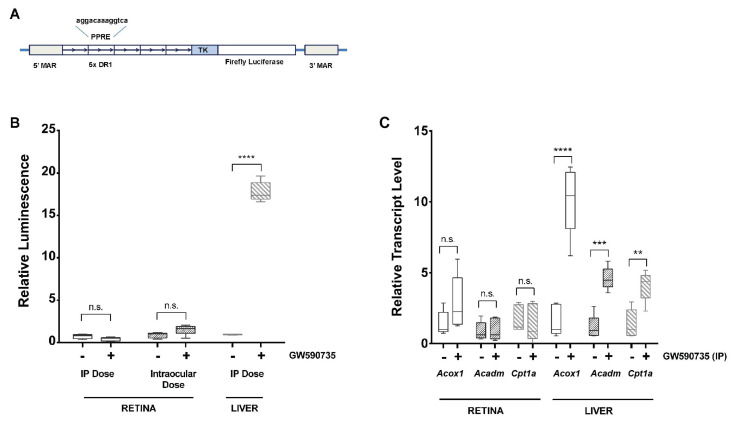
**Systemic or local delivery of PPAR-α agonists activates a PPRE-Luciferase reporter in liver but not retina.** (**A**) A construct consisting of five repeats of the peroxisome proliferator response element (PPRE) upstream of a basal thymidine kinase promoter (TK) driving the expression of firefly luciferase targeted to the nucleus with 5′ and 3′ matrix attachment regions (MARs) was used as a reporter to indicate activation of PPARα locally within tissues. (**B**) Relative luminescence was measured in retinal and liver tissue isolated from six-month-old mice that had been treated with the PPARα agonist GW590735. (**C**) No significant induction of reporter activity occurred in the retina following either intraperitoneal (IP) delivery of 10 mg/kg GW590735 or intraocular delivery of 2 μL of 500 nM GW590735. However, robust induction was noted in the liver following IP injection of GW590735. Intraperitoneal injection of GW590735 led to increased expression of PPARα-target genes *Acox1*, *Acadm*, and *Cpt1a* in the liver, but not in the retina, as assessed using qPCR. *n* = 5 per group. Statistical significance was determined using two-way ANOVA with Bonferroni correction (** *p* < 0.01, *** *p* < 0.001, **** *p* < 0.0001).

To further evaluate the ability of the retina to respond to PPARα agonists, a luciferase reporter assay was used to quantify PPARα activity in the retina and liver following treatment with the PPARα-selective agonist GW590735. Transgenic mice expressing the peroxisome proliferator response element (PPRE)-luciferase reporter received an intraperitoneal (IP) injection of GW590735 (10 mg/kg) or vehicle control at three months of age (Figure 6A). Retinas were harvested 24 h later, and luminance was measured as a proxy for PPARα activity. No induction of the reporter was observed in the retinas of mice treated with IP GW590735 (Figure 6B). Similarly, retinas isolated from animals receiving intravitreous injection of GW590735 (1 μL, 500 nM in DMSO/NS 50:50) or vehicle control showed no induction of the luciferase reporter (Figure 6B). However, liver isolated from mice that had received IP GW590735 did show robust reporter activation (Figure 6B).

To assess expression of endogenous PPARα targets, qPCR was conducted on retina and liver isolated from mice treated with IP GW590735. There was no significant increase in the expression of PPARα-target genes *Acox1*, *Acadm*, or *Cpt1* in the retina of mice treated with GW590735 (Figure 6C). However, all three genes showed significant increases in expression ranging from 5- to 10-fold in liver tissue (Figure 6C). Taken together with the luciferase assay, these data suggest that the PPARα pathway can be successfully induced by GW590735 in the liver, but not the retina, of non-diabetic mice.

## 4. Discussion

Our results confirm that oral fenofibrate alters metabolism in a mouse model of type 2 diabetes by altering circulating lipids, modulating gliosis and improving ERG abnormalities. Our findings are consistent with changes reported in other models of type 1 and type 2 diabetes, and models of retinopathy of prematurity [8,9,10,15,17,34,35]. These data lend further support to pursuing fenofibrate as a treatment for DR in patients. Models of type 2 diabetes, such as the *db/db* mouse and high fat diet, may be particularly useful in further exploring the action of fenofibrate as they more closely approximate the pathophysiology involved in the majority of patients with diabetes.

The ERG changes we report, namely, increased oscillatory potential implicit time and reduced b-wave amplitude, both indicate functional changes within the inner retina. These electroretinographic changes have long been noted in human patients with DR, and precede observable vascular changes in mice and in humans [8,26,36]. These data support a neurodegenerative process occurring in the inner retina prior to the onset of vascular compromise. With advancing technology allowing for the isolation and high-throughput sequencing of small numbers of cells, it should be possible in the future to better understand changes occurring within the Müller glia, vascular endothelial cells, pericytes, and the amacrine, horizontal, bipolar and ganglion neuronal subtypes that populate the inner retina.

Previously published data suggest that PPARα action locally in the retina is necessary for the protective effect of fenofibrate [13,14,15]. Therefore, our sequencing data suggesting that oral fenofibrate did not induce the expression of PPARα target genes in the retina or Müller glia were surprising. In addition, our luciferase reporter assay showed additional evidence that PPARα activity remained at baseline in the retina but was successfully induced in the liver with oral fenofibrate. Similar results have also been reported in the context of OIR, with fibrate treatment leading to the induction of PPARα target genes in the liver but not in the retina [37]. It is therefore plausible that oral fenofibrate acts as a PPARα agonist in the liver of patients with DR as well, which may drive the protective effects noted in the FIELD and ACCORD studies [5,6]. As these experiments were carried out in non-diabetic mice, it remains possible that local changes in PPARα may occur with fenofibrate treatment in the setting of diabetes. However, given the robust change in PPARα activation in the liver, even in non-diabetic animals, and the lack of induction in the retina, our data suggest that the liver is the primary target of fenofibrate.

We suspect that, rather than inducing PPARα directly in the retina, fenofibrate acts systemically via the liver to modulate circulating cytokines, growth factors and/or lipids that indirectly impact the retina. A reduction in diabetes-induced elevation of serum IL-1β, TNFα, VEGF, and Lp-LPA2 has been reported in diabetic patients in response to fenofibrate [38]. In a mouse model of diabetes, fenofibrate treatment was shown to increase serum Fgf21, resulting in local induction of oxidative stress response genes in the retina and kidney [37,39]. Such serum factors could result in the local changes in inflammation, apoptosis and oxidative stress response genes induced in the retina by fenofibrate as reported in the literature [17,34,35].

In summary, we report that fenofibrate modulates phenotypes of *db/db* mice including circulating triglycerides and cholesterol, retinal gliosis, and ERG changes. We found that fenofibrate did not induce the expression of PPARα-target genes in whole retina or Müller glia, but that PPARα activity was induced in the liver. Together, these data support a protective function of fenofibrate for DR, which may be mediated by activation of PPARα in the liver.

## Figures and Tables

**Table 1:** qPCR primer sequences.

Primer	Sequence
Mm_rho_f1	CTCTGCCAGCTTTCTTTGCT
Mm_rho_r1	GTCGTCATCTCCCAGTGGAT
Mm_GS_f1	CTGCCATACCAACTTCAGCA
Mm_GS_r1	TGTGGTACTGGTGCCTCTTG
Mm_Cpt1a_f1	TGGGAGAGAATTTCATCCACTT
Mm_Cpt1a_r1	TCCATCATGGCTTGTCTCAA
Mm_Acox1_f1	GATGTGACCCTTGGCTCTGT
Mm_Acox1_r1	GACTGCAGGGGCTTCAAGT
Mm_Acadm_f1	AGCTCTAGACGAAGCCACGA
Mm_Acadm_r1	GCGAGCAGAAATGAAACTCC
Mm_GAPDH_f1	TGCACCACCAACTGCTTAGC
Mm_GAPDH_r1	GGCATGGACTGTGGTCATGAG
Mm_L32_f1	TTCCTGGTCCACAATGTCAA
Mm_L32_r1	GGCTTTTCGGTTCTTAGAGGA

**Table 2:** Metabolic data of fenofibrate-treated mice in a model of type 2 diabetes.

	Non-Diabetic *db/m*		Diabetic *db/db*	
	Vehicle	Fenofibrate	Vehicle	Fenofibrate
*****n*****	10	8	10	10
**Body Weight, g, 3 months (at randomization)**	23.0 (1.2)	24.8 (1.4)	49.7 (1.1) ****	51.2 (2.1) ****
**Body Weight, g, 6 months (at assay)**	28.3 (1.3)	25.5 (0.7)	57.0 (2.4) ****	62.6 (0.9) ****
**Plasma Glucose (mg/dL) 3 months**	121.0 (6.8)	121.8 (8.8)	421.4 (29.9) ****	445.3 (28.1) ****
**Plasma Glucose (mg/dL) 6 months**	208.8 (9.1)	179.5 (8.1)	536.2 (33.8) ****	540.1 (32.9) ****
**Plasma Triglycerides (mg/dL) 6 months**	46.0 (1.4)	55.3 (5.1)	71.7 (4.5) ****	49.2 (2.2) ####
**Plasma Free Fatty Acids (mM) 6 months**	0.81 (0.04)	1.06 (0.09)	1.46 (0.04) ****	1.34 (0.07) *
**Plasma Cholesterol (mg/dL) 6 months**	49.6 (1.6)	84.2 (5.7) ##	103.2 (5.9) ****	127.6 (9.0) ***, #

Metabolic data for *db/db* and *db/+* mice are shown at baseline (three months of age), and at six months of age after three months of continuing a standard chow diet, or receiving a fenofibrate-supplemented diet. Body weight and plasma glucose levels were elevated in *db/db* mice relative to littermate controls at both timepoints and were not impacted by fenofibrate. Levels of plasma triglycerides, free fatty acids and cholesterol at the six-month timepoint are also shown. * = *p* < 0.05, *** = *p* < 0.001, **** = *p* < 0.0001, two-way ordinary ANOVA, compared to healthy control within same treatment group. # = *p* < 0.05, ## = *p* < 0.01, #### = *p* < 0.0001, two-way ordinary ANOVA, compared to genotype-matched control in the opposite treatment group.

**Table 3:** Tissue lipid content of fenofibrate-treated mice in a model of type 2 diabetes.

	Non-Diabetic *db/m*		Diabetic *db/db*	
	Vehicle	Fenofibrate	Vehicle	Fenofibrate
***n***	3	3	3	3
**Liver Cholesterol Content (mg/dL/g tissue) 6 months**	0.158 (0.016)	0.15 (0.005)	0.238 (0.015) *	0.255 (0.017) **
**Retinal Cholesterol Content (mg/dL/g tissue) 6 months**	0.123 (0.004)	0.145 (0.013)	0.137 (0.006)	0.127 (0.012)

At three months of age, *db/db* and *db/+* mice were randomized to receive fenofibrate-supplemented diets or to continue on a standard chow diet. At six months of age, cholesterol levels in the liver and retina were measured. Liver cholesterol was increased in *db/db* animals regardless of fenofibrate treatment, but retinal cholesterol levels were unchanged. * = *p* < 0.05, ** = *p* < 0.01, two-way ordinary ANOVA compared to healthy control within same treatment group.

## Data Availability

Raw RNA-Sequencing data and associated metadata are available at: http://www.ncbi.nlm.nih.gov/bioproject/689048. All other datasets from this study will be made available upon request.
